# Notes and News

**Published:** 1902-03-15

**Authors:** 


### Notes and News.

### HOSPITAL MEETINGS.

St. Mary's Hospital.—Annual Meeting, Friday, March 14th, at 4.30 p.m.

Royal Hospital for Diseases of the Chest.—Annual Meeting, Friday, March 14th, at 3 p.m.

Royal Eye Hospital.—Annual Meeting, Wednesday, March 19th, at 4 p.m.

University College Hospital.—Annual Meeting, Thursday, March 20th, at 4 p.m.

---

A statue is to be erected to the memory of Pasteur at Marne, where the great scientist passed the last years of his life.

---

It is probable that the present epidemic of small-pox will cause a reconsideration of all regulations relating to vaccination. It is to be hoped that the experience gained by medical officers of health will be collected for the general benefit. Dr. Hope, Medical Officer of Health for Liverpool, has already published a very practical and suggestive report.

---

Cases of plague have again broken out in Sydney. The epidemic is reported as being completely under control, the authorities stating very definitely that infection is to be looked for from rats, not from the patients. They take a very cheerful view of the disease, professing to regard it as more amenable than most epidemics. The number of patients has hardly increased at all since the plague appeared.

---

We regret to announce that, owing to continued ill-health, Mr. H. W. Custance, after nearly 30 years' devoted service, has resigned the Secretaryship of the Metropolitan Hospital Sunday Fund. His courtesy, whole-hearted interest in the work, and his punctual attendance to the duties of the office combined to increase the popularity of the Fund and its permanent usefulness. We hope that Mr. Custance may speedily be restored to health, and can assure him of the sincere sympathy of his many friends in the hospital world.

---

Sir Edmund Hay Currie, who has been one of the Honorary Secretaries of the Sunday Fund from its inception, has been elected Secretary, *vice* Mr. Custance, resigned. It is a very fortunate circumstance that Sir Edmund Currie should be willing to accept an office for which he is pre-eminently fitted by his experience and his great and successful labours in the field of charity.

---

The carcass of an animal that had suffered from acute general tuberculosis has been offered for sale in Aldgate.

---

An agitation for and against the legalisation of vivisection under definite restrictions is now taking place in Norway.

---

A clinical hospital for the University of Barcelona has been built by the Spanish Government at a cost of £200,000.

---

A report that small-pox has broken out severely in Antwerp and Brussels is accompanied by the statement that the disease has spread from London. Antwerp is an important port, and Belgium generally unpleasantly contiguous to other countries, so that it is to be hoped effective measures will be taken to arrest the epidemic.

---

The Rev. Hay Wilson, of St. Augustine's, Stepney, asks us to call attention to a leaflet advocating vaccination, entitled "Small-pox," which he has prepared to circulate especially amongst the poor. It is really an admirable statement of striking facts, clearly and concisely expressed, which can hardly fail to be convincing. It can be purchased from the Church Shop, 83 Commercial Road, for one halfpenny.

---

An interesting little function took place on Wednesday, March 5th, at the Station Hospital, Portsmouth, when Driver Arnold, Army Service Corps, was presented by Sir Evelyn Wood with the South African medal and five clasps. Arnold is a patient who has been some time in hospital, and is suffering from tuberculosis, but is not too ill to be otherwise than greatly pleased to receive his well-earned decoration from the hands of the distinguished General.

---

A gentleman who contributed £500 towards the cost of the hospital steamer *Queen Alexandra* which sailed from Great Yarmouth recently to take up work in the North Sea fishing fleets, being impressed with the urgent need there is for another boat of the same class, has intimated to the Royal National Mission to Deep Sea Fishermen his intention of giving £500 of the balance of £5,000 needed to build and equip a third hospital steamer. The society already has £7,000 in hand for the purpose.

---

The late Mr. Rathbone, of Liverpool, well deserved the name of philanthropist, and a striking instance of his thoughtfulness for others is shown in the request he left behind him that no friends should attend his funeral at the risk of their health. The attendance at a funeral in inclement weather is always associated with risk, even to those in sound health. The depressing circumstances of the funeral, the cold and damp which has often to be faced without the possibility of brisk movement as a protection, renders such occasions most dangerous. For the safety of the living, attendance at the graveside should be dispensed with for all in bad weather; but until this becomes the custom, it would be well that all should express the same wish that Mr. Rathbone bequeathed to his friends.

